# More opportunities more species: Pleistocene differentiation and northward expansion of an evergreen broad-leaved tree species *Machilus thunbergii* (Lauraceae) in Southeast China

**DOI:** 10.1186/s12870-021-03420-9

**Published:** 2022-01-17

**Authors:** Dengmei Fan, Shuqing Lei, Hua Liang, Qi Yao, Yixuan Kou, Shanmei Cheng, Yi Yang, Yingxiong Qiu, Zhiyong Zhang

**Affiliations:** 1 Laboratory of Subtropical Biodiversity, Jiangxi Agricultural University, Nanchang, Jiangxi China; 2 Systematic & Evolutionary Botany and Biodiversity Group, MOE Laboratory of Biosystem Homeostasis and Protection, College of Life Sciences, Zhejiang University, Hangzhou, Zhejiang China

**Keywords:** Population differentiation, Species diversity anomaly, Isolation-by-environment, Freezing temperature, Tropical niche conservatism, *Machilus thunbergii*, Southeast China

## Abstract

**Background:**

The broad continuum between tropical and temperate floras in Eastern Asia (EAS) are thought to be one of the main factors responsible for a prominent species diversity anomaly of temperate plants between EAS and eastern North America (ENS). However, how the broad continuum and niche evolution between tropical and temperate floras in EAS contributes to lineage divergence and species diversity remains largely unknown.

**Results:**

Population genetic structure, demography, and determinants of genetic structure [i.e., isolation-by-distance (IBD), isolation-by-resistance (IBR), and isolation-by-environment (IBE)] of *Machilus thunbergii* Sieb. et Zucc. (Lauraceae) were evaluated by examining sequence variation of ten low-copy nuclear genes across 43 populations in southeast China. Climatic niche difference and potential distributions across four periods (Current, mid-Holocene, the last glacial maximum, the last interglacial) of two genetic clusters were determined by niche modelling. North and south clusters of populations in *M. thunbergii* were revealed and their demarcation line corresponds well with the northern boundary of tropical zone in China of Zhu & Wan. The divergence time between the clusters and demographic expansion of *M. thunbergii* occurred after the mid-Pleistocene climate transition (MPT, 0.8–1.2 Ma). Migration rates between clusters were asymmetrical, being much greater from north to south than the reverse. Significant effects of IBE, but non-significant effects of IBD and IBR on population genetic divergence were detected. The two clusters have different ecological niches and require different temperature regimes.

**Conclusions:**

The north-south genetic differentiation may be common across the temperate-tropical boundary in southeast China. Divergent selection under different temperature regimes (possibly above and below freezing temperature in winter) could account for this divergence pattern. The broad continuum between tropical and temperate floras in EAS may have provided ample opportunities for tropical plant lineages to acquire freezing tolerance and to colonize the temperate regions during the late-Cenozoic global cooling. Our findings shed deeper insights into the high temperate plant species diversity in EAS.

**Supplementary Information:**

The online version contains supplementary material available at 10.1186/s12870-021-03420-9.

## Background

Owing to the general similarities in climate, vegetation types, and floristic composition between Eastern Asia (EAS) and eastern North America (ENA), a prominent species diversity anomaly of temperate plants between them has been bewildering generations of evolutionary biologists and ecologists [1–7]. This phenomenon is even more intricate in view of the fact that the contemporary floras of the two regions are largely derived from a single paleoflora, the “Boreotropical flora”, that was broadly distributed across the Northern Hemisphere during the early Cenozoic [8, 9]. The anomaly in species diversity has been associated with a few factors in EAS, such as high climatic and topographic heterogeneity, a stronger monsoon climate, and the broad continuum between tropical and temperate floras [3, 4, 7], which have stimulated many hypothesis-based phylogenetic and phylogeographic studies to elucidate the mechanisms underlying this prominent pattern [10–13].

The India-Eurasia collision since 55–40 million years ago (Ma) leads to the formation of the highest plateau of the world (Qinghai-Tibetan Plateau, QTP) and creates huge topographic and climatic heterogeneity in Eastern Asia [14]. Higher diversification rate has been demonstrated in a wide array of organisms on the QTP and adjacent regions [10, 15, 16]. The India-Eurasia collision also dramatically modified climates in EAS, a major climatic reorganization (from a planetary climate system to a monsoon-dominated pattern) occurred since the Early (23.0–16.0 Ma) to Middle Miocene (16.0–11.6 Ma) [17]. The mild monsoon climate, coupled with the complex topography of EAS, provided refugia for many ancient lineages, which has added species diversity as well as phylogenetic diversity to EAS [3, 12, 18]. Inexplicably, how the broad continuum between tropical and temperate floras contributes to the anomaly between EAS and ENA has received much less attention and seldom been tested explicitly.

According to the theory of niche conservatism, species will always inhabit environments that exhibit some similarities to those of their close relatives, for example, half of flowering plant families are strictly tropical and have not spread out of the tropics (tropical niche conservatism) [19, 20]. For a given tropical lineage, the only way to invade temperate climate regime is niche evolution, i.e., the expansion of niche breadth or specialization for new environment [21] because cold and highly seasonal climate especially freezing temperature in winter can cause lethal injuries in living plant tissues [22, 23] and represent a major factor limiting the dispersal of tropical plants into temperate regions (i.e., ecophysiological barrier) [24, 25]. However, species are not able to adapt to ecological conditions that they are never exposed to, the opportunities being afforded to individual species by their geographical location are essential for niche evolution [21].

It is widely known that a relatively continuous, homogenous flora with many tropical and subtropical taxa (the boreotropical flora) [8] covered almost the entire breadth of Eurasia and North America up to Arctic area during the early Cenozoic [2]. As climate cooled down during the middle and late Cenozoic towards the Pleistocene [26], most plants of the boreotropical flora at higher latitudes either migrated to lower latitudes or went extinct, only a subset of ancestrally tropical plant lineages succeeded to adapt to cold and seasonal climate [25]. Because EAS has a continuous latitudinal gradient of the forest vegetation even during the Pleistocene glaciations, whereas organisms in other parts of the Northern Hemisphere such as Europe and North America were heavily extirpated by thick ice sheets [27]. Therefore, the broad continuum between tropical and temperate floras throughout the late Cenozoic may have provided many more opportunities for the niche evolution of tropical plant lineages [25]. This hypothesis, we coin as “more opportunities more species”, offers a plausible explanation for the contribution of the continuum between tropical and temperate floras to the species richness anomaly of temperate plants between EAS and ENA, but has seldom been tested in any particular lineages.

*Machilus* Nees is a species-rich genus in Lauraceae containing ca. 100 evergreen tree or shrub species distributed in tropical and warm-temperate (subtropical) south Asia, with most species (ca. 82) occurring in south and southeast China [28]. Many species of this genus are dominant components of evergreen broad-leaved forests in tropical and warm-temperate regions of China [29], covering a latitudinal line that is called tropical-temperate divide in southwest America to Central America and straddling the northern boundary of the tropical zone in China recommended by Chinese meteorologists [30]. Across this divide, a shift in the tolerance of freezing temperature (niche evolution) has been reported in a few plant lineages in southwest America to Central America [31]. Recently, a few phylogeographic studies found a north-south break within the temperate-tropical divide of southeast China [32–35], implying niche evolution is needed for plants to cross this ecophysiological barrier. In this study, we selected *Machilus thunbergii* Sieb. et Zucc., a widespread and dominant components in tropical monsoon and warm-temperate evergreen broad-leaved forests [28] as a study system. Our previous phylogeographic study using chloroplast DNA sequences indicates that this species may have experienced extensive post-glacial range expansion [33]. Because a single maternally-inherited gene can hardly capture all major events in a species history, more insightful understandings of its evolutionary history should be gained by using multi-locus sequence data. In this study, we used 10 nuclear loci to analyze the phylogeographic structure and determined the potential drivers of spatial genetic variation of *M. thunbergii*. In particular, we addressed the question of whether or not a clear north-south differentiation can be observed within this species. Specifically, based on multi-locus sequence data, we applied an approximate Bayesian computation (ABC) procedure to test competing scenarios of population demographic history that has potentially contributed to the contemporary distribution of the species. In addition, we used landscape genetic approaches to examine the relative contributions of geography and ecology to genetic divergence. Together, these analyses would contribute to a better understanding of how the broad continuum and niche evolution between tropical and temperate floras in EAS contribute to lineage divergence and thus species diversity.

## Results

### Sequence variation and neutrality test of nuclear loci

Ten low-copy nuclear genes were sequenced for all 211 individuals from 43 populations. The total aligned length was 3130 bp, with loci ranging from 221 to 389 bp (Table S1). A 6-bp indel locating in a non-coding region of MT159 was detected and excluded from subsequent analyses. A total of 186 segregating sites, including 52 singletons, were found from 10 nuclear genes.

Average genetic diversity parameters, such as total nucleotide diversity, silent nucleotide diversity, non-synonymous nucleotide diversity, and haplotype diversity, were relatively low at species level (*π*_t_ = 0.00356, *π*_s_ = 0.00663, *π*_a_ = 0.00410, *H*_d_ = 0.565). As for the two population clusters (see below), average genetic diversity was much higher in south cluster (*π*_t_ = 0.00439, *π*_s_ = 0.00751, *π*_a_ = 0.00336, *H*_d_ = 0.655) than in north cluster (*π*_t_ = 0.00230, *π*_s_ = 0.00456, *π*_a_ = 0.00162, *H*_d_ = 0.418) (Table S2). In each cluster, negative and mostly non-significant Tajima’s *D* values and Fu and Li’s D* and F* values were detected at the majority of 10 nuclear loci (Table S2). There was non-significant deviation from neutrality in each cluster using multilocus Hudson-Kreitman-Aguadé tests. In addition, at both cluster and species levels, MFDM tests rejected the likelihood of natural selection acting on individual loci (results not shown).

### Population genetic structure and demographic history

**Fig. 1:**
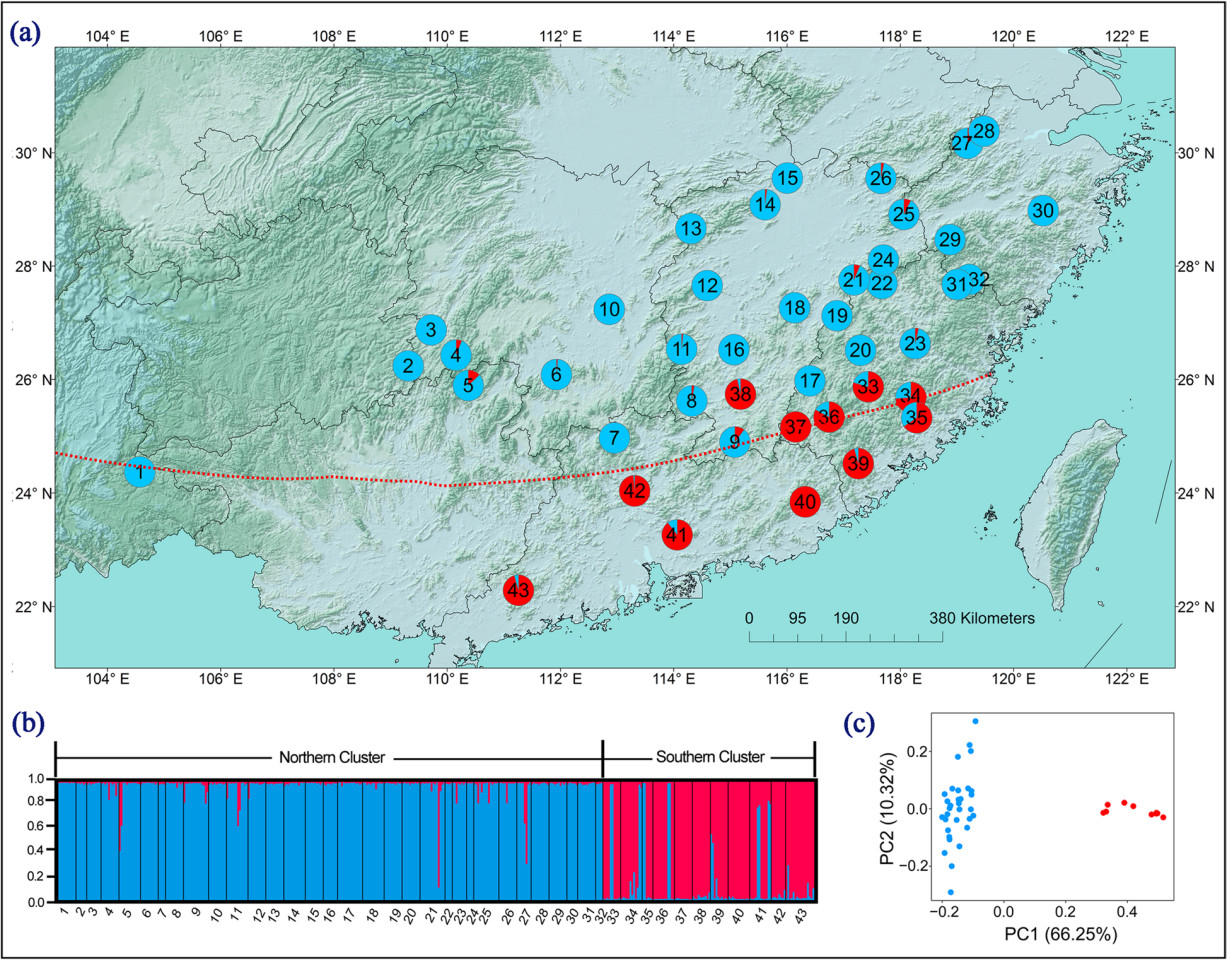
**a** Colour-coded grouping of the 43 *Machilus thunbergii* populations according to STRUCTURE with the most likely group number *K* = 2. The colored pies were drawn on the China map which was downloaded from https://www.webmap.cn/;**b** histogram of the STRUCTURE assignment for all individuals at *K* = 2; **c** principle component analysis on pairwise genetic differentiation of populations. The red dotted line shows the northern boundary of tropical zone in broad sense [30, 36]

The LnP(*D*) and Δ*K* statistics inferred from the Bayesian clustering algorithm (STRUCTURE) showed the most likely number of clusters for the entire dataset was *K* = 2 (Fig. S1). The two clusters clearly correspond to the geographic distribution: the north cluster (N) comprised of populations 1–32, and the south cluster (S) contained populations 33–43 (Fig. 1a and b). Two population clusters was also found in PCA (Fig. 1c). The demarcation line of the two clusters corresponds well with the northern boundary of tropical zone in China of Zhu & Wan [30] with a few exceptions possibly due to microhabitat differences.

**Fig. 2:**
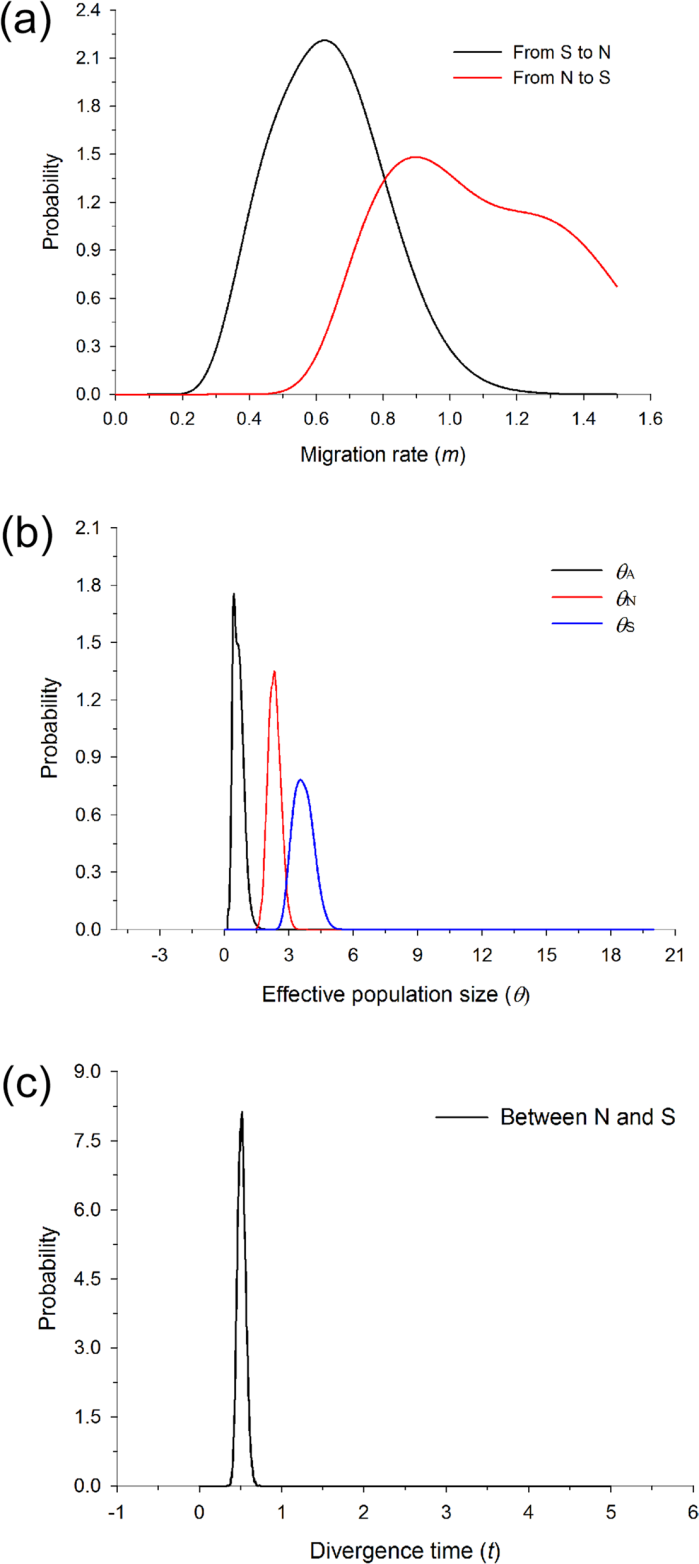
Posterior probability distributions of (**a**) the migration rate (m) between two clusters (N and S) from both directions, (**b**) effective population size (*θ*) for each cluster (N, S and all populations), and (**c**) divergence time (*t*) between two clusters (N and S) estimated by IM model

The divergence time between clusters N and S was estimated to 0.58 million years ago [95% highest posterior density (HPD) intervals: 0.41–0.71 Ma] using IMa2 (Table S3). This time is slightly older than the divergence time estimated in ABC modeling [0.46 Ma, 95%CI (0.085–0.9 Ma)]. Migration rates (*m*) estimated under the IM model using IMa2 were asymmetrical, with much higher gene flow from N to S (1.468) than in the reverse direction (0.456) (Fig. 2, Table S3).

**Fig. 3:**
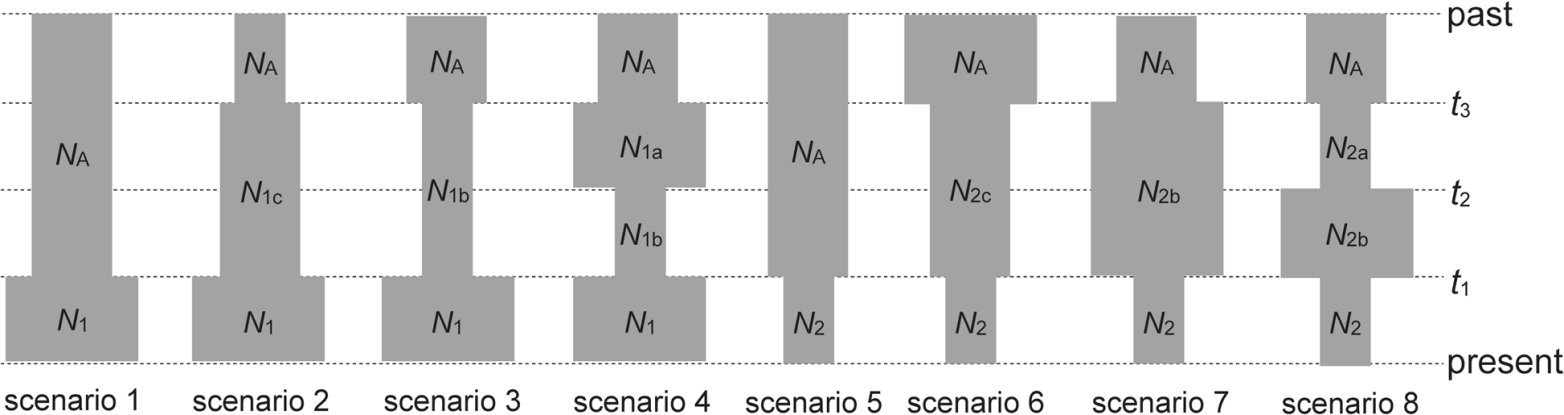
Eight assumed demographic scenarios tested for clusters N, S, and A in DIYABC. *N*_1_ and *N*_2_ represent current population sizes, and *N*_A_ represents the ancestral population size. *N*_1a_, *N*_1b_, *N*_1c_, *N*_2a_, *N*_2b_ and *N*_2c_ represent population sizes between the ancestral population and the current population. *t*_1_, *t*_2_ and *t*_3_ represent times of population changes

Among eight potential demographic scenarios in ABC modeling (Fig. 3), *M. thunbergii* as a whole, clusters N and S all conformed to scenario 2 (91.03, 61.58, 65.46% posterior probability, respectively; Table S4, Fig. S2), which indicates stepwise population expansions. One expansion time (*T*_3_) was estimated at 0.68 Ma (95% CI: 0.25–0.98 Ma), 0.52 Ma (95% CI: 0.12–0.94 Ma) and 0.46 Ma (95%CI: 0.09–0.93 Ma), respectively, and the other (*T*_1_) was at 0.10 Ma (95% CI: 0.02–0.19 Ma), 0.15 Ma (95% CI: 0.02–0.28 Ma) and 0.07 Ma (95% CI: 0.01–0.14 Ma), respectively (Table 1, Fig. S3). The LAMARC analysis also suggested that the two clusters have undergone changes in population size. For clusters N and S, the estimated *g* values were 3005.49 and 7234.10, respectively, suggestive of population growth. The EBSP simulations indicated the two clusters and *M. thunbergii* as a whole all experienced continuous recent expansions (Fig. S4).

**Table 1:** Posterior estimates of demographic parameters for the best models of population demography in Fig. 3 revealed by Approximate Bayesian Computation

Cluster (best model)	Parameter	*N*_A_	*N*_1_	*N*_1c_	*T*_1_ (years)	*T*_1_ (generations)	*T*_3_ (years)	*T*_3_ (generations)
N (scenario 2)	Mean	4.10 × 10^4^	1.03 × 10^6^	3.7 × 10^5^	1.47 × 10^5^	1.47 × 10^4^	5.20 × 10^5^	5.20 × 10^4^
	Median	3.89 × 10^4^	1.02 × 10^6^	3.45 × 10^5^	1.42 × 10^5^	1.42 × 10^4^	5.17× 10^5^	5.17× 10^4^
	95% CI	0.71–8.29 × 10^5^	0.30–1.80 × 10^6^	0.72–7.38 × 10^5^	0.19–2.83 × 10^5^	0.19–2.83 × 10^4^	1.21–9.43 × 10^5^	1.21–9.43 × 10^4^
S (scenario 2)	Mean	4.19 × 10^4^	2.74 × 10^6^	2.28 × 10^5^	7.31 × 10^4^	7.31 × 10^3^	4.55 × 10^5^	4.55 × 10^4^
	Median	3.85 × 10^4^	2.64 × 10^6^	2.09 × 10^5^	6.90 × 10^4^	6.90 × 10^3^	4.16 × 10^5^	4.16 × 10^4^
	95% CI	0.84–8.85 × 10^4^	0.72–4.72 × 10^6^	0.58–4.55 × 10^5^	0.11–1.41 × 10^5^	0.11–1.41 × 10^4^	0.86–9.25 × 10^5^	0.86–9.25 × 10^4^
A (scenario 2)	Mean	5.81 × 10^4^	3.08 × 10^6^	1.01 × 10^6^	1.02 × 10^5^	1.02 × 10^4^	6.80 × 10^5^	6.80 × 10^4^
	Median	5.68 × 10^4^	2.92 × 10^6^	0.98 × 10^6^	0.99 × 10^5^	0.99 × 10^4^	7.20 × 10^5^	7.20 × 10^4^
	95% CI	0.06–1.12 × 10^5^	0.92–5.64 × 10^6^	0.27–1.80 × 10^6^	0.20–1.88 × 10^5^	0.20–1.88 × 10^4^	2.47–9.78 × 10^5^	2.47–9.78 × 10^4^

*N*_A_, ancestral population size; *N*_1_, current population sizes; *N*_1c_, population size between the ancestral population and the current population; *T*_1_, *T*_3_, population expansion time

### IBD, IBE and IBR

**Fig. 4:**
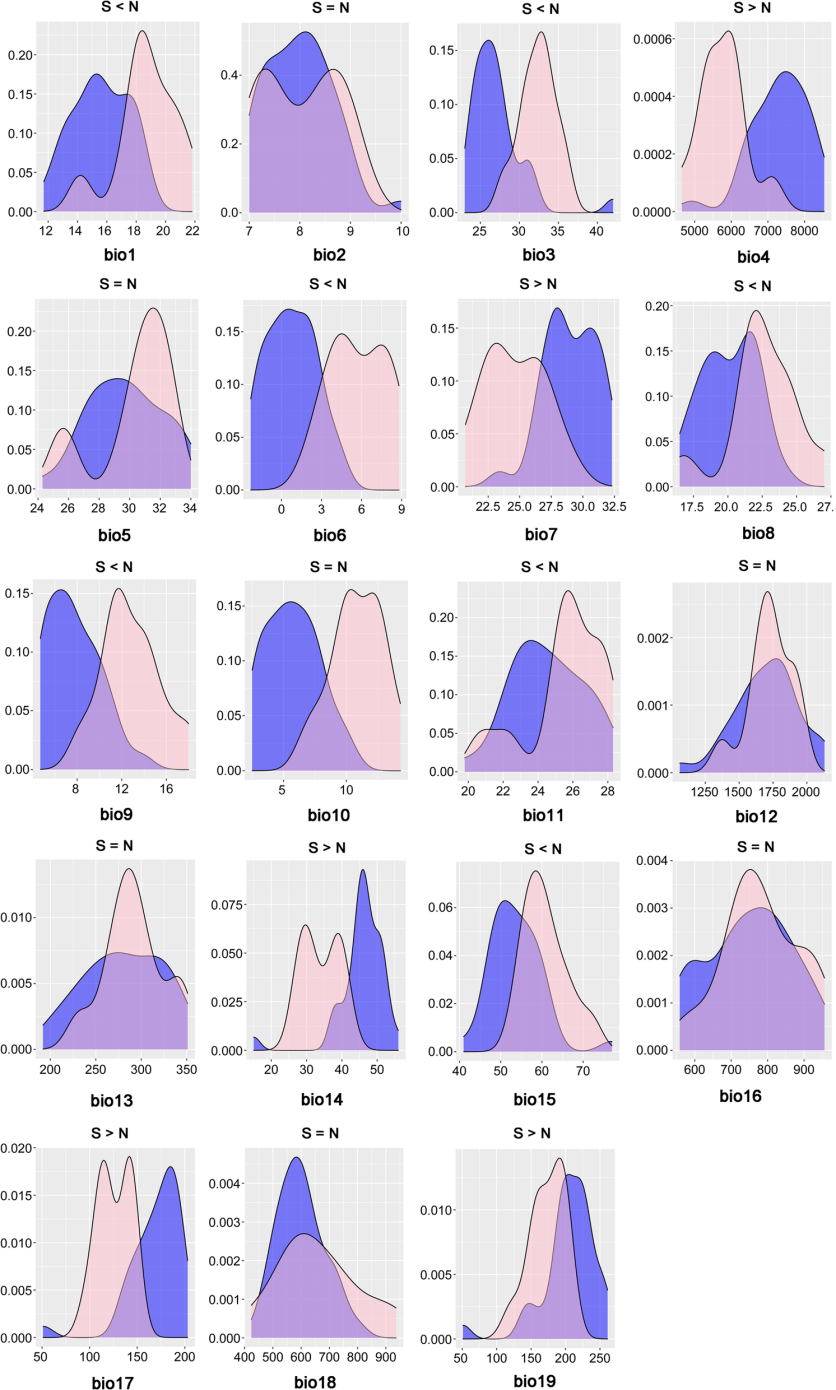
Kernel density plots for 19 environmental parameters of the northern (blue curves) and southern (pink curves) clusters of *M. thunbergii*. Differentiation among groups was evaluated by a nonparametric Kruskal-Wallis multiple-range test, and the results are indicated in each plot. A lack of significant difference (at the *P* < 0.05 level) is indicated by an equal sign, while significant differences are coded as either higher or lower

The multiple matrix regression with randomization (MMRR) analysis revealed significant effects of isolation by environment (IBE) on population genetic divergence (*F*_ST_) (*β*_IBE_ = 0.482, *P* = 0.001) but non-significant effects of isolation by distance (IBD) (*β*_IBD_ = − 0.037, *P* = 0.636) and isolation by resistance (IBR) (*β*_IBR_ = − 0.083, *P* = 0.405). The principal component analysis found clear climatic differentiation among two genetic clusters (N and S) in the first two principal components (PC1: 51.63%, PC2: 28.13%) (Fig. S5). Frequency distributions of N and S clusters for each environmental variable are shown in kernel density plots (Fig. 4). Nonparametric Kruskal-Wallis multiple-range tests suggested that 12 of the 19 bioclimatic variables, except for Bio2 (mean diurnal range), Bio5 (max temperature of warmest month), Bio10 (mean temperature of warmest quarter), Bio12 (annual precipitation), Bio13 (precipitation of wettest month), Bio16 (precipitation of wettest quarter), and Bio18 (precipitation of warmest quarter), distinguished significant differences between two clusters (Fig. 4).

### Ecological niche modeling and niche identity tests

**Fig. 5:**
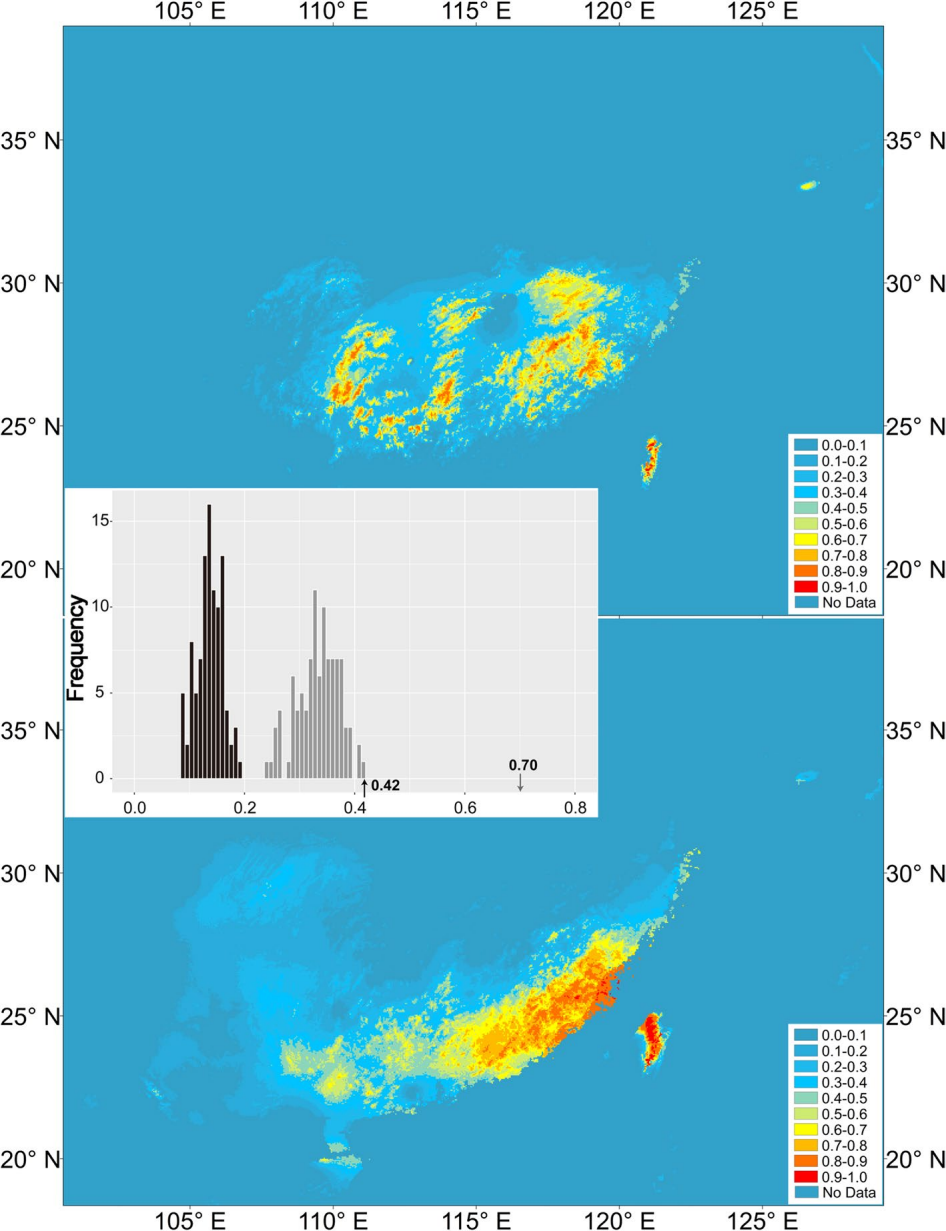
Current potential distributions of two *Machilus thunbergii* clusters (N and S) predicted by MAXENT, and identity tests results between two groups. The black bars indicate the null distributions of *D*, while the grey bars indicate *I*. Arrows indicate values of *D* (black) and *I* (grey) in actual MAXENT runs

ENMs were constructed for the two *M. thunbergii* clusters to predict their current potential distributions and the model was then projected to past scenarios (Fig. 5). Nine bioclimatic variables (Bio2: mean diurnal range, Bio5: max temperature of warmest month, Bio6: min temperature of coldest month, Bio7: temperature annual range, Bio8: mean temperature of wettest quarter, Bio13: precipitation of wettest month, Bio15: precipitation seasonality, Bio18: precipitation of warmest quarter, Bio19: precipitation of coldest quarter) were retained with *r* < 0.7 in each pair. In the MAXENT climate models, both groups had high AUC values, indicating that the climate model predicted occurrence well (northern cluster test data AUC = 0.986, SD = 0.007 and southern cluster test data AUC = 0.989, SD = 0.899). Observed measures of niche similarity (*D* and *I*) were higher than null distributions for N vs. S, suggesting high niche differentiation between N and S (Fig. 5). A jackknife test of the AUC values for both groups indicated the most effective climate variables for predicting occurrence were minimal temperature of coldest month (northern cluster AUC = 0.952; southern cluster AUC = 0.940) and precipitation of coldest quarter (northern cluster AUC = 0.968; southern cluster AUC = 0.955) (Table S5).

The predicted present-day, LIG, LGM and MH distributions were nearly identical for the northern group (Fig. S6). For southern group, clear range expansions were predicted for the LGM and MH model relative to the present day (Fig. S6).

## Discussion

### Late Quaternary divergence history of *Machilus thunbergii*

In this study, we found a clear north-south population differentiation in an evergreen broadleaved tree species *Machilus thunbergii* belonging to Lauraceae in southeast China. This pattern has been repeatedly observed in other plant species (e.g., *Ficus pumila* (Moraceae), *Schima superba* (Theaceae), *Lindera aggregata* (Lauraceae), *Oreocharis auricula* (Gesneriaceae)) [32–35]. Similarly, in North and Middle America (Mexico and Central America), a north-south phylogeographic break across the so-called tropical-temperate divide was also reported at the similar latitudes (ca. 15–33°N) in live oaks (*Quercus* series *Virentes*) [31]. Although the number of study cases is still limited, a common north-south differentiation of plant species straddling the tropical-temperate divide is beginning to emerge in southeast China as well as in North and Middle America. Interestingly, such a steep transition across the tropical-temperate divide has also been observed in several functional traits such as seed size and plant height [37, 38], indicating that there might be a genetically-grounded switch in plant strategy between temperate and tropical zones [38, 39].

The tropical niche conservatism theory predicts that tropical plant lineages may have spread to temperate regions recently only after a shift from tropical to temperate niche had happened during the late-Cenozoic global cooling [19, 20]. In line with the prediction, we found that the north-south separation of *M. thunbergii* happened between 0.58–0.46 Ma and demographic expansions after 0.52 Ma for each population cluster. In addition, we also detected that much lower genetic diversity in north cluster than that in south cluster, conforming the prediction that temperate lineages are often recently derived from lineages in tropical regions, leading to shallower phylogenetic divergences among temperate lineages than among tropical lineages [21].

Although conforming to theoretical prediction, the separation time of north-south groups is substantially younger than the ages (~ 30–40 Ma) of many temperate plant clades that originated in the tropics [24] when temperate regions expanded [40, 41]. This difference may be simply explained by that intraspecific divergence is often much shallower than interspecific diversification. However, only after the climate changes since the late Pliocene, is it possible for plant species in southeast China to adapt to temperate climate, because the temperate region was confined to relatively high latitudes for most periods of Cenozoic, with tropical climate dominating central to south China until late Miocene [42]. However, global temperatures began to drop sharply during the Pliocene, about 3 Mya, and then, in the Pleistocene, underwent violent fluctuations at intervals of about 100,000 years [26]. During the Last Maximum Glaciation (LGM), for instance, the mean annual temperature dropped by c. 4–6 °C [43] and the evergreen broad-leaved forests was forced to the south of 24°N in China [44].

Note that the time of population differentiation and demographic expansion of *M. thunbergii* is well after the mid-Pleistocene climate transition (MPT, 0.8–1.2 Ma) [45], a period marked by an increase in the severity of glaciations and the emergence of the ~ 100-kyr glacial cycles [46]. It was reported that the earliest and largest glaciation in China (Wangkun glaciation, 700–500 ka) [47] occurred shortly after the MPT. The ice sheet during Wangkun glaciation was 18 times larger than the present ice sheet on the Qinghai-Tibetan Plateau [48]. Demographic expansions have been reported in several plant species of central to southeast China shortly after the MPT [12, 49]. It is possible that the largest glaciation in China might have exposed the ancestral tropical populations of *M. thunbergii* to cold climate and the tolerance of freezing temperature might be acquired by some descendants during 0.58–0.46 Ma, producing genetically and possibly geographically distinct populations. In the subsequent warm and humid interglacial (0.50–0.46 Ma) [47], the north populations which had adapted to temperate climate could have experienced strong demographic expansion as suggested in our previous study [33]. Surprisingly, in contrast to the demographic stability of the south populations of another Lauraceae species (*Lindera aggregata*) [34] in the same region, southern populations of *M. thunbergii* could have also experienced demographic expansion posterior to the expansion of north populations (0.46 Ma vs 0.52 Ma). This event led to secondary contacts of the diverging lineages and a pattern of asymmetrical gene flow towards south populations, a phenomenon that frequently occurs from the local to the invading lineages [50, 51].

### Quantifying the effects of landscape, geography and ecology in spatial genetic divergence of *Machilus thunbergii*

Physical barriers such as mountains, rivers, inhospitable habitats or even man-made buildings are the most common and straightforward factors responsible for pronounced population differentiation [10, 52–56]. In southeast China, there are several major mountains stretching from west to east or from southeast to northeast (e.g., Nanling Mountains, Wuyi-Xianxia Mountains) which might serve as physical barriers to gene flow. For example, the uplifting of Wuyi-Xianxia Mountains during the Pliocene has been inferred as the causes for the separation of *Ficus pumila* populations to the north and south of the mountains [32]. However, we did not find significant isolation-by-resistance in *M. thunbergii* that account for landscape heterogeneity, suggesting the major mountains in southeast China can not impede gene flow of *M. thunbergii* effectively. Indeed, the Nanling Mountains and Wuyi-Xianxia Mountains, with peaks rarely exceeding 2000 m above sea level, have been frequently proved to be glacial refugia and/or dispersal corridors, rather than to be physical barriers in previous phylogeographic studies [49, 57, 58]. In addition, we did not find significant isolation-by-distance, indicating that genetic drift alone can not produce the north-south differentiation of *M. thunbergii*, either.

On the contrary, significant effect of isolation-by-environment was detected by the multiple matrix regression with randomization (MMRR) analysis (β_IBE_ = 0.482, *P* = 0.001). This result indicates that divergent selection associated with environmental heterogeneity, especially changes in climatic regimes, could be the driving force responsible for the population differentiation of *M. thunbergii.* Many mechanisms such as (natural selection against immigrants, sexual selection against immigrants, reduced hybrid fitness and biased dispersal) can generate IBE [56], direct evidence for local adaptation to different climatic conditions should be gained through common garden experiments [59] or through genomic scanning of the loci underlying natural selection [60]. Although this study did not compare the performance (such as freezing tolerance) of the two provenances of *M. thunbergii* in common gardens and the genomic loci responsible for local adaptation can not be deciphered due to the paucity of nuclear loci, several lines of evidence suggest that the north-south population differentiation could have been mainly caused by local adaptation to different climatic conditions.

First, it is generally assumed that loci under divergent selection and those tightly physically linked to them may exhibit stronger differentiation than neutral regions with weak or no linkage to such loci. However, divergent selection can also increase genome-wide neutral differentiation by reducing gene flow, thus promoting divergence via the stochastic effects of genetic drift [61]. In addition, neutral loci (i.e., loosely linked loci) that are far along a chromosome from a selected site may be influenced by selection because hitchhiking effects can extend a considerable distance from the selected locus [62–64]. Therefore, although the loci we examined may not be the targets of divergent selection, the elevated genetic differentiation between the north and south clusters suggests that population divergence could be caused by hitchhiking effects on loosely linked loci.

Second, climatic adaptation is a highly important component in the evolution of plants with temperature potentially the most important selective agent over an altitudinal/latitudinal cline [65]. Freezing temperatures can cause lethal injuries in living plant tissues [22] and have long been considered a principal restriction on the spreading out of tropics for many tropical pant lineages [25]. This implies that migration from tropical to temperate regions is not easy which may greatly restructure metabolic, morphological, defensive and phenological traits. For a particular tropical plant lineage, an opportunity opposing to divergent selective regimes of temperature is required for generating and maintaining genetic adaptation to temperate climate. The distribution range of *M. thunbergii* spans the tropical-temperate boundary suggested by Zhu & Wan [30] (Fig. 1a), south of which there is no frost damage in winter [30, 36]. Although the exact freezing temperature of *M. thunbergii* remains to be determined, it is very likely that the north and south populations of *M. thunbergii* could have been experiencing different freezing regimes in winter. In addition, the importance of minimal temperature of coldest month in predicting the distribution of *M. thunbergii* in niche modeling (Table S4) and significant differences of many temperature variables (Fig. 4) in nonparametric Kruskal-Wallis tests also support the strong role of different temperature regimes in driving population divergence of *M. thunbergii*.

Third*,* to the south of Zhu & Wan’s Line, the frequency of tropical genera can reach 60% of the regional flora, however, the frequency decrease sharply to the north of the line [66]. For example, *Ficus microcarpa*, a dominated tree species in tropical forests of southeast China, reach south Jiangxi and central Fujian (25–26°N) [67]. However, transplanted individuals to the north of 25°N always suffer from freezing damage and can not survive the winter outdoors (personal observation). These facts strongly suggest that there is an ecophysiological barrier in southeast China that is associated with the freezing temperature of plants to prevent tropical plants from invasion into temperate zones. Successful colonizers such as the north cluster of *M. thunbergii* should have gained a new ecological niche via genetic modifications in their evolutionary history.

### Implications for understanding plant species diversity anomaly between EAS and ENA

Although population differentiation can not be translated into speciation, the genetic divergence among populations within a species will ultimately lead to speciation or incomplete speciation [68, 69]. Thus, the common phenomenon of north-south differentiation observed in *M. thunbergii* and other plant species in southeast China may have important implications for understanding plant species diversity anomaly between EAS and ENA.

First, while there are a wealth of tropical species in southeast Asia, they *per se* may contribute little to the temperate species pool of East Asia, because the freezing temperature represents an ecophysiological barrier for them to colonize the temperate zone. For instance, the distribution range of many tropical lineages of southeast Asia, such as fig trees, seldom extends to the north of 25° N [66]. Second, EAS is characterized by a continuous landmass without being interrupted by sea. This physiographical setting may translate into an amplitude of opportunities for tropical plant lineages to adapt to temperate climate in EAS during the late-Cenozoic global climate cooling. However, such continuum is interrupted in North America by the Gulf of Mexico (and in Europe by the Mediterranean and the Sahara Desert) [3]. Because of the narrow continuum between temperate and tropical floras, north-south genetic differentiation across the tropical-temperate divide of North America has rarely been documented [31], suggesting far less opportunities for the niche evolution and speciation of tropical plant lineages in North America. Third, the commonality and recency of north-south population differentiation imply that the recruitment of tropical plant species into species pool of EAS through adaptive evolution is an ongoing process which might further contribute to the species diversity anomaly between EAS and ENA in the future. Therefore, southeast China is a key area deserving a high conservation priority not only for its high plant species diversity [70, 71], but also for the evolutionary processes that generate the diversity.

## Conclusions

Using multi-locus phylogeographic and landscape genetic approaches, this study revealed a north-south genetic differentiation in *M. thunbergii* and the demarcation line of north-south population clusters corresponds well with the northern boundary of tropical zone in China of Zhu & Wan [30]. Such a divergence pattern may be common across the temperate-tropical boundary in southeast China that could be associated with divergent selection under different temperature regimes (possibly above and below freezing temperature in winter). The results have profound implications for understanding the prominent species diversity anomaly of temperate plants between EAS and ENS in that the broad continuum between tropical and temperate floras in EAS may have provided ample opportunities for tropical plant lineages to acquire freezing tolerance and to colonize the temperate regions during the late-Cenozoic global cooling. Future studies are needed to gain direct evidence for local adaptation to different climatic conditions through common garden experiments or through genomic scanning of the loci underlying natural selection.

## Methods

### Plant material

In total, leaf tissues from 211 individuals of *Machilus thunbergii* were sampled from 43 wild populations in Southeast China (Table S6, Fig. 1) and were immediately dried with silica gel. Sampled individuals were spaced at least 50 m for each population. All voucher specimens were deposited in the Herbarium of Lushan Botanical Garden of the Chinese Academy of Sciences (Table S6).

### DNA extraction, amplification and sequencing

Total genomic DNA was extracted from silica-dried leaves using a modified cetyltrimethylammonium bromide (CTAB) method [72]. Ten low-copy nuclear genes (MT02, MT04, MT15, MT33, MT55, MT57, MT96, MT115, MT159 and MT164) were developed from transcriptome sequences of *M. thunbergii* (Table S1) by the method of our previous studies [73, 74]. Transcriptome sequencing was performed on the Illumina NextSeq 500 platform (Illumina, San Diego, CA, USA) at Personal Biotechnology (Shanghai, China). The sequences of transcriptome unigenes were deposited at figshare (https://figshare.com/articles/dataset/Unigene_fa/15090174).

The PCR reaction systems and procedures were the same as those in our previous study [33]. Different annealing temperatures were set for each locus (Table S1). Sequencing reactions were carried out with the corresponding primers in both directions commercially by Sangon Biotech Co., Ltd. (Shanghai, China). All sequences were edited with Sequencher 4.14 (GeneCodes Corporation, Ann Arbor, MI, USA), aligned using Bioedit 7.2 [75] and refined manually in Mega 5.05 [76]. All DNA sequences were deposited in the NCBI GenBank database (accession Nos: MW856287–MW856301, MW872423–MW872637).

### Nucleotide diversity and neutrality tests

For each nuclear locus, the sequences were phased using the phase algorithm in Dnasp 5.10 [77]. Then we used Dnasp 5.10 [77] to estimate the basic population genetic parameters, i.e., the number of segregating sites (*S*), nucleotide diversity (*π*), Watterson’s parameter (*θ*_w_) [78], the number of haplotypes (*N*_h_), haplotype diversity (*H*_d_) and the minimum number of recombinant events (*R*_m_). We also calculated Tajima’s *D* [79] and Fu and Li’s *D* and *F* [80] to test the neutral evolution of each locus. The multilocus Hudson–Kreitman–Aguadé test (HKA) [81] was further performed in Dnasp 5.10 to assess the fit of data to neutral equilibrium using *Machilus villosa* (Roxb.) Hook. f. as an outgroup. Finally, we used the maximum frequency of derived mutations (MFDM) test [82] to examine the likelihood of natural selection acting on individual loci.

### Population genetic structure

Population structure was assessed using Structure 2.3.4 [83] with the admixture model. Twenty independent runs were conducted for each number of clusters (*K*) from 1 to 10 with burn-in setting to 20,000 and Markov chain Monte Carlo (MCMC) run length to 200,000. The LnP(D) [84] and Δ*K* statistics [85] were used to estimate the most likely number of clusters. Graphics were visualized by Distruct 1.1 [86].

### Population divergence and demography

We used the isolation by migration (IM) model to estimate gene flow and divergence time between genetic clusters in the IMa2 software package [87, 88]. The run sets were the same with our previous study [73]. Parameters were estimated based on a mean mutation rate of 3.4×10^− 9^ per site per year, which is estimated in *Lindera aggregata* (Lauraceae) [34], another species in Lauraceae. Average generation time was set to 10 years as in previous study [33].

The genetic divergence time between genetic clusters was also tested using ABC approach implemented in Diyabc 2.1.0 [89]. In addition, eight demographic scenarios (Fig. 3) were tested using ABC for cluster N, cluster S and all populations (A), respectively: (1) one expansion (t1), (2) two expansions (t3, t1), (3) one contraction (t3) and one expansion (t1), (4) one expansion (t3), one contraction (t2), and then another expansion (t1), (5) one contraction (t1), (6) two contractions (t3, t1), (7) one expansion (t3) and one contraction (t1), and (8) one contraction (t3), one expansion (t2), and another contraction (t1). Another Bayesian method was also implemented by Lamarc 2.1.8 to estimate the population size changes [90]. The performance settings for DIYABC and Lamarc followed our previous study [73]. DIYABC selects the optimized rate from a given mutation range between 10^− 7^ and 10^− 9^. All summary statistics were chosen to test the posterior probability parameters. To ensure statistically robust results, at least 8, 000, 000 simulated datasets were generated for each scenario. In order to identify the best-supported scenario, we estimated posterior probabilities [with 95% confidence intervals (CIs)] of each scenario using logistic regression of 1% of simulated data sets closest to the observed data.

Furthermore, we used extended Bayesian skyline plots (EBSP) analysis in Beast 2.5.1 [91] to infer the temporal changes in the effective population size (*N*_e_) changes of the Structure clusters. We used the default substitution model and the substitution rate of 3.4 × 10^− 9^ per site per year [34]. We set the MCMC to 20,000,000 steps with sampling every 20,000 steps, and set the burn-in at 20%.

### Determinants of population genetic structure

Isolation by distance (IBD), isolation by environment (IBE) and isolation by resistance (IBR) has been considered as the main forces for driving the population structure across a species’ range [92]. Isolation by distance correlates the genetic differences and geographical distances between populations or subgroups [93]. IBE describes the accumulation of genetic divergence when environmental differences between sampled populations increase [56]. Resistance distances are often the outcome of physically isolating barriers (such as mountains or rivers) that can structure spatial genetic divergence [94]. To understand the determinants of population genetic structure, we firstly generated four distance matrices with all populations included. Genetic distances (*F*_ST_) among each pair of populations were calculated using Arlequin 3.01 [95]; A geographical distance matrix was calculated using the GPS coordinates of populations. We used the sum option in the Ressistance And Habitat Calculator tool in Arcmap 10.8.1 to calculate resistance values from raster layers of elevation, roads, water bodies and rivers. Then the pairwise resistance distance matrix were generated using Circuitscape 4.0 [94]. Nineteen bioclimatic variables were extracted from current climatic data with 30 arc-seconds resolution (http://www.worldclim.org) and used as proxies of environmental gradients. A climatic distance matrix was constructed using population pairwise Euclidean distances across all 19 bioclimatic variables. Then, after standardizing the four distance matrices by *Z* scores, we quantified the contributions of geographical distance, resistance distance and environmental distance to genetic distance using the ‘Multiple Matrix Regression with Randomization (MMRR)’ function in R 3.6.3 [96].

In this study, we detected significant isolation-by-environment (see Results). To further detect which climatic variable is associated with the genetic differentiation between the two clusters, we conducted a principal component analysis (PCA) on climate data (19 climate variables) obtained from Worldclim (http://www.worldclim.org) located within the sampling areas of the two groups. In addition, we used nonparametric Kruskal–Wallis multiple-range test [97] to examine the difference of each environmental variable between the two clusters. The distribution of each environmental variable was displayed in kernel density plots. We performed these analyses and drew graphical illustrations in R 3.6.3.

### Ecological niche modeling

Current potential distributions for northern and southern clusters were predicted using ecological niche modeling (ENM) in Maxent 3.3.4 [98] with the settings as follows: replicates = 20; type = subsample; maximum iterations = 5000; random test points = 25. Nineteen bioclimatic layers with a 2.5 arc minute resolution were downloaded from the WorldClim database. For the mid-Holocene (MH) and last glacial maximum (LGM), the last interglacial (LIG), we employed paleoclimate data based on MIROC-ESM model. The geographical coordinates of 32 sampling sites from northern cluster and 11 from southern cluster were used for developing niche models. The pairwise Pearson correlations (*r*) among the bioclimatic variables were examined to avoid multicollinearity, and we excluded one of the variables in each pair with *r* > 0.7. The AUC (area under the receiver operating characteristic curve) values were used to measure model accuracy, and the model with values above 0.9 was considered better discrimination. We also conducted the Jackknife tests to identify the contribution of each climatic variable. The niche differences between the two groups were measured by niche overlap and identity tests in Enmtools 1.4.4 using statistics of Schoener’s *D* and standardized Hellinger distance (*I*) [99].

## Supplementary Information

**Additional file 1.**

## Data Availability

The sequences of transcriptome unigenes were deposited at figshare (https://figshare.com/articles/dataset/Unigene_fa/15090174). All DNA sequences of 10 nuclear loci have been deposited in the NCBI GenBank database under accession numbers MW856287–MW856301, MW872423–MW872637. The datasets supporting the conclusions of this article are included within the article and its additional files. The datasets used and/or analyzed during the current study are available from the authors on reasonable request (Dengmei Fan, dmf.625@163.com).

## Abbreviations

EAS: Eastern Asia
ENA: Eastern North America
QTP: Qinghai-Tibetan Plateau
ABC: Approximate Bayesian computation
MPT: Mid-Pleistocene climate transition
