# Supplementary material for: More opportunities more species: Pleistocene differentiation and northward expansion of an evergreen broad-leaved tree species Machilus thunbergii (Lauraceae) in Southeast China
Source: BMC Plant Biol. 2022 Jan 17;22:35. doi: 10.1186/s12870-021-03420-9 (PMC8762935; doi:10.1186/s12870-021-03420-9)
Supplement: Supplementary file 1 — Additional file 1. [file 12870_2021_3420_MOESM1_ESM.docx]

**Table S1** Basic information for 10 self-developed nuclear low copy gene primers in *Machilus thunbegii.*

| Locus | Unigene | Sequence(5'-3') | Annealing temp(°C) | PCR product (bp) |
| --- | --- | --- | --- | --- |
| MT2 | c1007_g1 | R: GACTGGGAATAGACTGCTTA | 50 | 333 |
|  |  | F: AGGTGGATTGATAGTTGGA |  |  |
| MT4 | c10005_g1 | R: AGGTCTGATTTGGGTGTA | 50 | 389 |
|  |  | F: ATGGGATTGACTTTGGAG |  |  |
| MT15 | c10357_g | R: TTCTCCCATCTCACTGTTTC | 50 | 279 |
|  |  | F: AGGGCTTCTTTCTGTCGG |  |  |
| MT33 | c1146 | R: CAAACAGCCTCCCTATGAACT | 50 | 386 |
|  |  | F: TATCGGCGAAACCCTAATC |  |  |
| MT55 | c27611_g1 | R: TCGCCAACATCTGAACATAG | 50 | 259 |
|  |  | F: AACCTCCCAAGAACACTC |  |  |
| MT57 | c27603_g2 | R: GTATGTTGACCGCTTCCT | 55 | 309 |
|  |  | F: ACTGCTCATTTGGTGCTT |  |  |
| MT96 | c12246 | R: AAGAGCAGAACGGCACCTA | 50 | 299 |
|  |  | F: GCAATCCACCCATACCATC |  |  |
| MT115 | c33818_g1 | R: CAACACCCTCCCTCTTAT | 50 | 221 |
|  |  | F: ATGAACTGCCTGATGACT |  |  |
| MT159 | c20655 | R: GGTTGGTGTTGCCTGTTT | 55 | 355 |
|  |  | F: CTATCTTGCCTTGTTCATCG |  |  |
| MT164 | c20749 | R: ATAACCAGCCCAACCAAT | 50 | 300 |
|  |  | F: TAAAGCCAAGCCAATCCT |  |  |

**Table S2** Genetic parameters of 10 nuclear loci.

|  |  | Total | | | | | |  | Silent sites | | |  | Nonsynonymous sites | | |  | Haplotype diversity | |  | Neutrality tests | | |
| --- | --- | --- | --- | --- | --- | --- | --- | --- | --- | --- | --- | --- | --- | --- | --- | --- | --- | --- | --- | --- | --- | --- |
| group | Locus | *N* | *L* | *R*_m_ | *S* | *θ*_w_ | *π* |  | *S*s | *θ*_s_ | *π*_s_ |  | *S*_a_ | *θ*_a_ | *π*_a_ |  | *N*_h_ | *H*_d_ |  | Tajama’s *D** | Fu &Li’s *F** | Fu &Li’s *D** |
| North cluster | MT2 | 211 | 333 | 1 | 4 | 0.00573 | 0.00045 |  | 2 | 0.00435 | 0.00062 |  | 10 | 0.00611 | 0.00040 |  | 12 | 0.127 |  | -2.140* | -2.674* | -2.269 |
|  | MT4 | 211 | 389 | 1 | 20 | 0.00572 | 0.00165 |  | 5 | 0.00987 | 0.00105 |  | 9 | 0.00467 | 0.00182 |  | 16 | 0.546 |  | -1.708 | -2.672* | -2.535* |
|  | MT15 | 211 | 279 | 1 | 7 | 0.00456 | 0.00103 |  | 4 | 0.01034 | 0.00219 |  | 4 | 0.00301 | 0.00073 |  | 11 | 0.210 |  | -1.622 | -1.922 | -1.591 |
|  | MT33 | 211 | 386 | 2 | 11 | 0.00495 | 0.00149 |  | 11 | 0.01791 | 0.00567 |  | 2 | 0.00111 | 0.00007 |  | 16 | 0.313 |  | -1.710 | -1.271 | -0.651 |
|  | MT55 | 211 | 259 | 1 | 12 | 0.00920 | 0.00237 |  | 2 | 0.00467 | 0.00039 |  | 13 | 0.01087 | 0.00309 |  | 16 | 0.473 |  | -1.809* | -0.660 | -0.253 |
|  | MT57 | 211 | 309 | 0 | 8 | 0.00309 | 0.00169 |  | 2 | 0.00532 | 0.00022 |  | 4 | 0.00255 | 0.00205 |  | 5 | 0.485 |  | -0.870 | -1.240 | -1.126 |
|  | MT96 | 211 | 299 | 0 | 8 | 0.00319 | 0.00024 |  | 4 | 0.00865 | 0.00045 |  | 2 | 0.00142 | 0.00017 |  | 7 | 0.071 |  | -1.781* | -3.302** | -3.283** |
|  | MT115 | 211 | 221 | 1 | 13 | 0.01007 | 0.00695 |  | 10 | 0.03205 | 0.02378 |  | 4 | 0.00375 | 0.00210 |  | 15 | 0.791 |  | -0.745 | 0.270 | 0.829 |
|  | MT159 | 211 | 355 | 2 | 15 | 0.00637 | 0.00231 |  | 10 | 0.01884 | 0.00902 |  | 4 | 0.00244 | 0.00018 |  | 15 | 0.437 |  | -1.532 | -2.590* | -2.535 |
|  | MT164 | 211 | 300 | 2 | 21 | 0.01059 | 0.00486 |  | 1 | 0.00240 | 0.00220 |  | 19 | 0.01292 | 0.00561 |  | 27 | 0.723 |  | -1.392 | -1.388 | -0.986 |
|  | Average | 211 | 313 | 1 | 12 | 0.00635 | 0.00230 |  | 5 | 0.01144 | 0.00456 |  | 7 | 0.00489 | 0.00162 |  | 14 | 0.418 |  | -1.368 | -1.035 | -1.073 |
| South cluster | MT2 | 211 | 333 | 1 | 4 | 0.00225 | 0.00122 |  | 0 | 0 | 0 |  | 3 | 0.00225 | 0.00130 |  | 6 | 0.291 |  | -0.884 | 0.399 | 0.927 |
|  | MT4 | 211 | 389 | 1 | 20 | 0.00962 | 0.00302 |  | 2 | 0.00466 | 0.00180 |  | 18 | 0.01098 | 0.00336 |  | 15 | 0.642 |  | -1.948* | -1.911 | -1.490 |
|  | MT15 | 211 | 279 | 1 | 7 | 0.00470 | 0.00434 |  | 4 | 0.01228 | 0.00535 |  | 3 | 0.00265 | 0.00417 |  | 10 | 0.770 |  | -0.175 | 0.852 | 1.181 |
|  | MT33 | 211 | 386 | 3 | 11 | 0.00535 | 0.00448 |  | 11 | 0.02113 | 0.01769 |  | 0 | 0 | 0 |  | 17 | 0.837 |  | -0.415 | -1.180 | -1.311 |
|  | MT55 | 211 | 259 | 0 | 12 | 0.00867 | 0.00283 |  | 0 | 0 | 0 |  | 12 | 0.01182 | 0.00385 |  | 12 | 0.470 |  | -1.750 | -2.097 | -1.765 |
|  | MT57 | 211 | 309 | 2 | 8 | 0.00484 | 0.00581 |  | 1 | 0.00308 | 0.00028 |  | 7 | 0.00528 | 0.00716 |  | 11 | 0.722 |  | 0.473 | -0.121 | -0.405 |
|  | MT96 | 211 | 299 | 1 | 8 | 0.00501 | 0.00106 |  | 5 | 0.01273 | 0.00295 |  | 3 | 0.00251 | 0.00045 |  | 7 | 0.177 |  | -1.866* | -1.079 | -0.405 |
|  | MT115 | 211 | 221 | 1 | 13 | 0.01101 | 0.00459 |  | 9 | 0.03401 | 0.01592 |  | 4 | 0.00442 | 0.00133 |  | 12 | 0.557 |  | -1.537 | -2.318* | -2.175 |
|  | MT159 | 211 | 355 | 3 | 15 | 0.00804 | 0.00620 |  | Na | Na | 0.02228 |  | Na | Na | 0.00109 |  | 21 | 0.844 |  | -0.760 | -1.537 | -1.598 |
|  | MT164 | 211 | 300 | 5 | 21 | 0.01310 | 0.00714 |  | 2 | 0.00565 | 0.00128 |  | 19 | 0.01521 | 0.00880 |  | 27 | 0.873 |  | -1.301 | -0.938 | -0.463 |
|  | Average | 211 | 311 | 2 | 13 | 0.00782 | 0.00439 |  | 4 | 0.01169 | 0.00751 |  | 8 | 0.00661 | 0.00336 |  | 15 | 0.655 |  | -0.781 | -1.001 | -0.937 |

*N*, sample size; *L*, length in base pairs; *R*_m_, minimum number of recombinant events; *S*, number of segregating sizes; *θ*_w_, Watterson’s parameter (Watterson 1975); *π*, nucleotide diversity (Nei 1987; Nei & Li 1979); *N*_h_, number of haplotypes; *H*_d_, Nei’s haplotype diversity; *D*, Tajima’s *D* statistic; *D** and *F**, Fu and Li’s *D** and Fu and Li’s *F**; Na, failed to be computed for lack of enough variation.

**Table S3** Maximum-likelihood estimates (MLE) and 95% highest posterior density (HPD) intervals of demographic parameters from IMa2 multiloci analyses.

| Parameter | *θ*_1_ | *θ*_2_ | *θ*_A_ | *m*_1_ | *m*_2_ | *t* | *N*_1_ | *N*_2_ | *N*_A_ | *T* |
| --- | --- | --- | --- | --- | --- | --- | --- | --- | --- | --- |
| MLE | 2.227 | 3.440 | 0.448 | 0.456 | 1.468 | 0.6175 | 5.23×10^5^ | 8.08×10^5^ | 1.05×10^5^ | 0.58×10^6^ |
| HPD95Lo | 1.819 | 2.795 | 0.240 | 0.263 | 0.813 | 0.4375 | 4.27×10^5^ | 6.57×10^5^ | 0.56×10^5^ | 0.41×10^6^ |
| HPD95Hi | 2.834 | 4.246 | 0.791 | 0.747 | 1.999 | 0.7525 | 6.66×10^5^ | 9.97×10^5^ | 2.04×10^5^ | 0.71×10^6^ |

*θ*_1_ and *N*_1_, effective population size of cluster N; *θ*_2_ and *N*_2_, effective population size of cluster S; *θ*_A_ and *N*_A_, ancestral effective population size. *m*_1_, population migration rate from cluster S to cluster N; *m*_2_, population migration rate from cluster N to cluster S;. *θ*, *m* and *t* are scaled by the mutation rate, while *N*_1_, *N*_2_ and *T* are scaled by individuals or years.

**Table S4** Posterior probabilities and credibility intervals of eight scenarios in Fig. 3 used in Approximate Bayesian Computation modeling.

| Scenario | Posterior probability (Credibility interval) | | |
| --- | --- | --- | --- |
|  | N | S | A |
| 1 | 0.1870 [0.1559,0.2180] | 0.2220 [0.2107,0.2334] | 0.0406 [0.0348,0.0464] |
| 2 | 0.6158 [0.5954,0.6363] | 0.6546 [0.6416,0.6676] | 0.9103 [0.8997,0.9209] |
| 3 | 0.1319 [0.0890,0.1748] | 0.0411 [0.0369,0.0452] | 0.0427 [0.0367,0.0488] |
| 4 | 0.0639 [0.0292,0.0986] | 0.0758 [0.0702,0.0813] | 0.0063 [0.0054,0.0072] |
| 5 | 0.0000 [0.0000,0.0000] | 0.0000 [0.0000,0.0000] | 0.0000 [0.0000,0.0000] |
| 6 | 0.0000 [0.0000,0.0000] | 0.0000 [0.0000,0.0000] | 0.0000 [0.0000,0.0000] |
| 7 | 0.0000 [0.0000,0.0000] | 0.0061 [0.0026,0.0095] | 0.0000 [0.0000,0.0000] |
| 8 | 0.0014 [0.0000,0.0382] | 0.0005 [0.0000,0.0040] | 0.0002 [0.0000,0.0006] |

**Table S5** Environmental variables and their importance parameters used in distribution predicting of northern and southern clusters of *Machilus thunbergii.*

| Variable | Description | *PC* (%) | *PI* (%) | *RTG_W_* | *RTG_O_* | *TG_W_* | *TG_O_* | *AUC_W_* | *AUC_O_* |
| --- | --- | --- | --- | --- | --- | --- | --- | --- | --- |
| Northern group |  |  |  |  |  |  |  |  |  |
| Bio2 | Mean diurnal range | 1.0292 | 1.1530 | 3.2625 | 1.8380 | 3.3231 | 1.8706 | 0.9867 | 0.9399 |
| Bio5 | Max temperature of warmest month | 0.1379 | 0.0490 | 3.2788 | 0.4443 | 3.3258 | 0.5485 | 0.9865 | 0.7837 |
| Bio6 | Min temperature of coldest month | 5.8445 | 88.4528 | 3.1760 | 2.0028 | 3.2742 | 2.1356 | 0.9848 | 0.9520 |
| Bio7 | Temperature annual range | 0.0655 | 0.6186 | 3.2773 | 1.4652 | 3.3211 | 1.6686 | 0.9865 | 0.9346 |
| Bio8 | Mean temperature of wettest quarter | 5.5436 | 3.2633 | 3.1958 | 0.5932 | 3.2224 | 0.8050 | 0.9858 | 0.8438 |
| Bio13 | Precipitation of wettest month | 0.0401 | 0.2105 | 3.2781 | 1.4283 | 3.3440 | 1.5478 | 0.9868 | 0.9149 |
| Bio15 | Precipitation seasonality (coefficient of variation) | 3.3710 | 1.6277 | 3.2558 | 1.3266 | 3.3086 | 1.4582 | 0.9864 | 0.9164 |
| Bio18 | Precipitation of warmest quarter | 18.9179 | 2.5595 | 3.2546 | 1.6826 | 3.3229 | 1.8581 | 0.9859 | 0.9433 |
| Bio19 | Precipitation of coldest quarter | 65.0501 | 2.0656 | 3.2195 | 2.4263 | 3.2987 | 2.5344 | 0.9855 | 0.9684 |
| Southern group |  |  |  |  |  |  |  |  |  |
| Bio2 | Mean diurnal range | 34.9172 | 1.4556 | 2.5519 | 1.5580 | 2.9181 | 1.6223 | 0.9883 | 0.9331 |
| Bio5 | Max temperature of warmest month | 0.0199 | 0 | 2.5586 | 0.0631 | 2.9205 | 0.0943 | 0.9886 | 0.6803 |
| Bio6 | Min temperature of coldest month | 18.5145 | 56.0169 | 2.2954 | 1.3714 | 2.5910 | 1.657 | 0.9837 | 0.9403 |
| Bio7 | Temperature annual range | 32.6023 | 26.2534 | 2.3947 | 1.2225 | 2.8578 | 1.2771 | 0.9881 | 0.9038 |
| Bio8 | Mean temperature of wettest quarter | 0.0605 | 0 | 2.5577 | 0.1898 | 2.9357 | 0.2563 | 0.9888 | 0.7298 |
| Bio13 | Precipitation of wettest month | 0.0023 | 0.0008 | 2.5584 | 0.8800 | 2.9190 | 1.2879 | 0.9886 | 0.9117 |
| Bio15 | Precipitation seasonality (coefficient of variation) | 10.1742 | 14.8901 | 2.4836 | 0.4983 | 2.9211 | 0.7687 | 0.9880 | 0.8121 |
| Bio18 | Precipitation of warmest quarter | 0.2893 | 0.0228 | 2.5557 | 0.7283 | 2.9418 | 1.1318 | 0.9892 | 0.9179 |
| Bio19 | Precipitation of coldest quarter | 3.4198 | 1.3604 | 2.5227 | 1.2769 | 2.8027 | 1.7780 | 0.9870 | 0.9548 |

*PC*, percent contribution; *PI*, permutation importance; *RTG_W_*, regularized training gain without the variable; *RTG_O_*, regularized training gain with only the variable; *TG_W_*, test gain without the variable; *TG_O_*, test gain with only the variable; *AUC_W_*, AUC without the variable; *AUC_O_*, AUC with only the variable.

**Table S6** Sample location, sample size, voucher specimens and genetic diversity of 10 nDNA sequences in 43 sampled populations of *Machilus thunbergii.*

| Region/  Population ID | Location | | Latitude  (°N) | | Longitude  (°E) | | | N | Voucher specimen No. | |
| --- | --- | --- | --- | --- | --- | --- | --- | --- | --- | --- |
| Northern |  | |  | |  | | |  |  | |
| 1 | Jinzhongshan, Jiangxi | | 24.37 | | 104.57 | | | 5 | LBG00148325 | |
| 2 | Liping, Guizhou | | 26.24 | | 109.31 | | | 3 | LBG00148349 | |
| 3 | Huitong, Hunan | | 26.87 | | 109.72 | | | 4 | LBG00148348 | |
| 4 | Suining, Hunan | | 26.43 | | 110.16 | | | 5 | LBG00148350 | |
| 5 | Mao'er'shan, Guangxi | | 25.89 | | 110.37 | | | 6 | LBG00148351 | |
| 6 | Shuangpai, Hunan | | 26.09 | | 111.93 | | | 5 | LBG00148346 | |
| 7 | Yizhang, Hunan | | 24.97 | | 112.95 | | | 2 | LBG00148347 | |
| 8 | Chongyi, Jiangxi | | 25.62 | | 114.32 | | | 5 | LBG00148326 | |
| 9 | Dingnan, Jiangxi | | 24.90 | | 115.16 | | | 7 | LBG00148327 | |
| 10 | Hengshan, Hunan | | 27.24 | | 112.86 | | | 5 | LBG00148345 | |
| 11 | Jinggangshan, Jiangxi | | 26.53 | | 114.14 | | | 6 | LBG00148328 | |
| 12 | Fenyi, Jiangxi | | 27.66 | | 114.60 | | | 5 | LBG00148329 | |
| 13 | Tonggu, Jiangxi | | 28.66 | | 114.30 | | | 5 | LBG00148330 | |
| 14 | Yongxiu, Jiangxi | | 29.08 | | 115.61 | | | 6 | LBG00148309 | |
| 15 | Lushan, Jiangxi | | 29.55 | | 116.01 | | | 5 | LBG00148310 | |
| 16 | Taihe, Jiangxi | | 26.53 | | 115.06 | | | 4 | LBG00148331 | |
| 17 | Shicheng, Jiangxi | | 26.02 | | 116.34 | | | 7 | LBG00148332 | |
| 18 | Le'an, Jiangxi | | 27.26 | | 116.13 | | | 6 | LBG00148333 | |
| 19 | Lichuan, Jiangxi | | 27.12 | | 116.88 | | | 5 | LBG00148311 | |
| 20 | Jiangle, Fujian | | 26.52 | | 117.30 | | | 5 | LBG00148312 | |
| 21 | Zixi, Jiangxi | | 27.76 | | 117.18 | | | 7 | LBG00148315 | |
| 22 | Wuyishan, Fujian | | 27.75 | | 117.68 | | | 2 | LBG00148314 | |
| 23 | Nanping, Fujian | | 26.63 | | 118.26 | | | 4 | LBG00148313 | |
| 24 | Shangrao, Jiangxi | | 28.11 | | 117.70 | | | 2 | LBG00148316 | |
| 25 | Sanqingshan, Jiangxi | | 28.91 | | 118.06 | | | 7 | LBG00148317 | |
| 26 | Fuliang, Jiangxi | | 29.55 | | 117.66 | | | 5 | LBG00148318 | |
| 27 | Qingliangfeng, Zhejiang | | 30.17 | | 119.20 | | | 4 | LBG00148320 | |
| 28 | TianMushan, Zhejiang | | 30.37 | | 119.47 | | | 5 | LBG00148319 | |
| 29 | Jiulongshan, Zhejiang | | 28.47 | | 118.88 | | | 3 | LBG00148322 | |
| 30 | Pan'an, Zhejiang | | 28.98 | | 120.52 | | | 5 | LBG00148321 | |
| 31 | Jinzifeng, Zhejiang | | 27.68 | | 119.01 | | | 5 | LBG00148323 | |
| 32 | Qingyuan, Zhejiang | | 27.75 | | 119.21 | | | 2 | LBG00148324 | |
| Southern |  |  | |  | |  |  | |  |  |
| 33 | Yong'an, Fujian | | 25.87 | | 117.43 | | | 5 | LBG00148344 | |
| 34 | Dehua, Fujian | | 25.68 | | 118.19 | | | 6 | LBG00148343 | |
| 35 | Yongchun, Fujian | | 25.32 | | 118.29 | | | 3 | LBG00148342 | |
| 36 | Shangkang, Fujian | | 25.34 | | 116.74 | | | 6 | LBG00148341 | |
| 37 | Wuping, Fujian | | 25.16 | | 116.15 | | | 5 | LBG00148340 | |
| 38 | Ganxian, Jiangxi | | 25.74 | | 115.18 | | | 5 | LBG00148334 | |
| 39 | Nanjing, Fujian | | 24.52 | | 117.26 | | | 5 | LBG00148339 | |
| 40 | Fengshun, Guangdong | | 23.85 | | 116.32 | | | 6 | LBG00148335 | |
| 41 | Boluo, Guangdong | | 23.27 | | 114.06 | | | 6 | LBG00148336 | |
| 42 | Yingde, Guangdong | | 24.19 | | 113.41 | | | 4 | LBG00148337 | |
| 43 | Xinyi, Guangdong | | 22.29 | | 111.26 | | | 8 | LBG00148338 | |


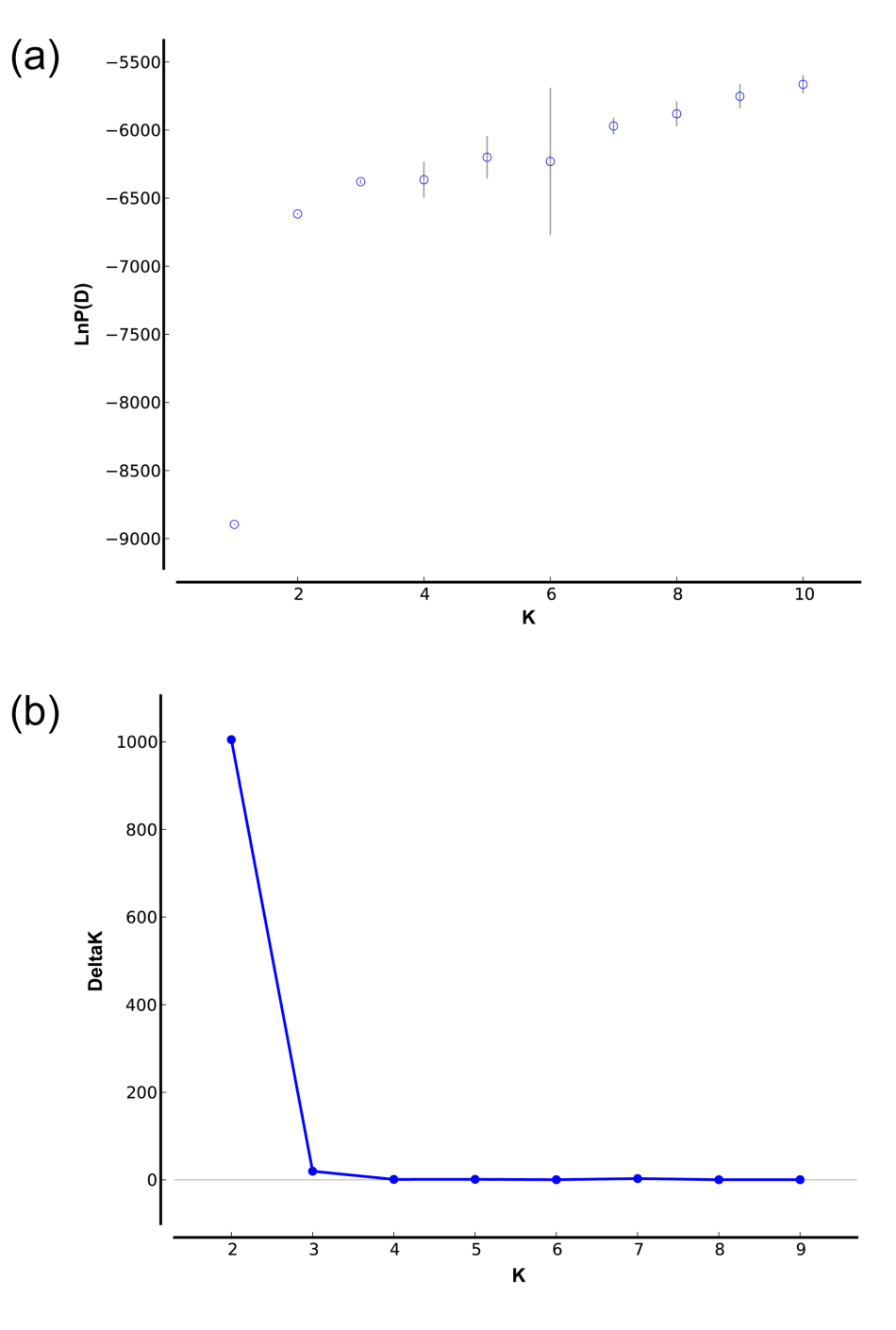


**Fig. S1** LnP(*D*) (a) and Delta-*K* (b) values from the STRUCTURE analysis on 43 *Machilus thunbergii* populations with predefined group number K=1-10. Standard deviations of LnP(*D*) obtained from 10 independent runs for each group number are also shown.

**
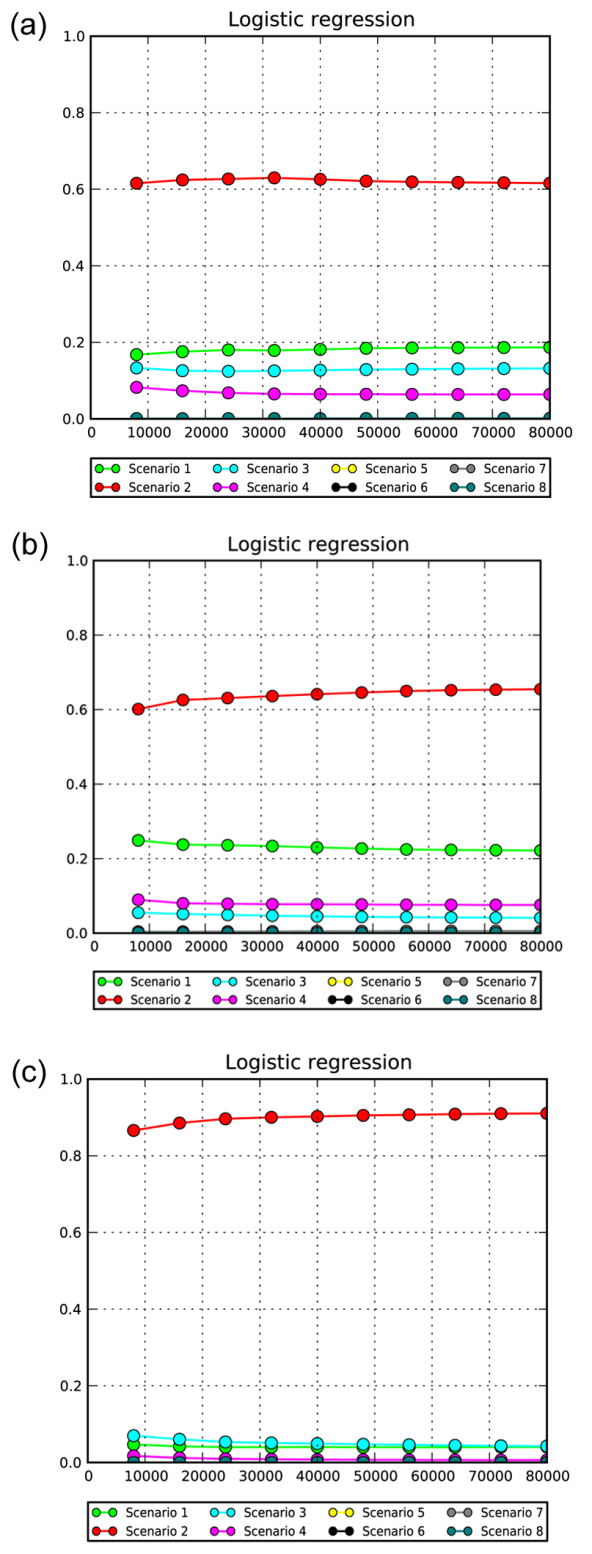
**

**Fig. S2** Posterior probabilities of eight scenarios used in Fig. 3 for (a) N, (b) S, and (c) A estimated using logistic regression of 1% of the closest datasets in DIYABC.


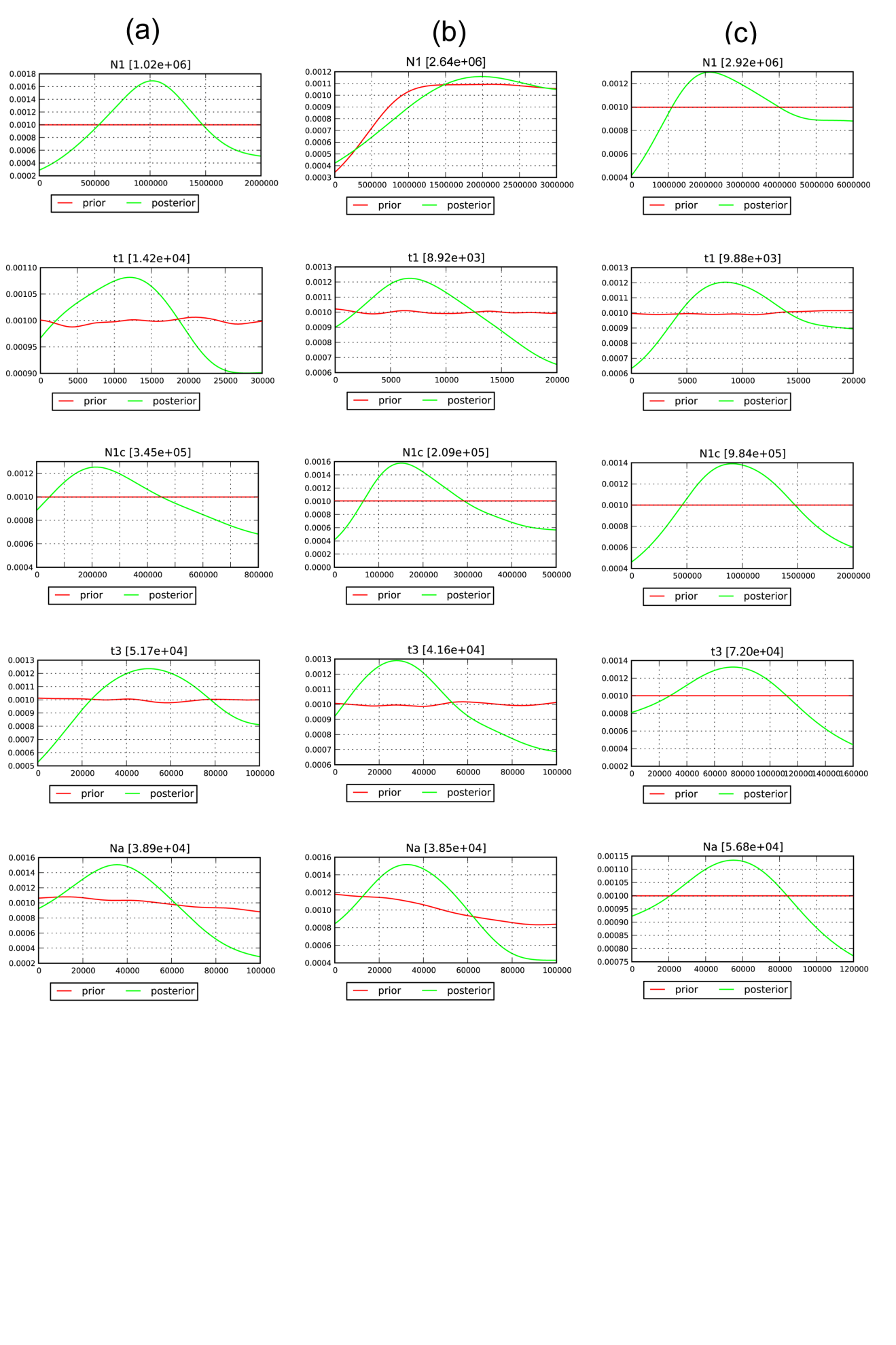


**Fig. S3** Prior and posterior distributions of demographic parameters for (a) N (scenario 2), (b) S (scenario 2), and (c) A (scenario 2) in Fig. 3 estimated using DIYABC.


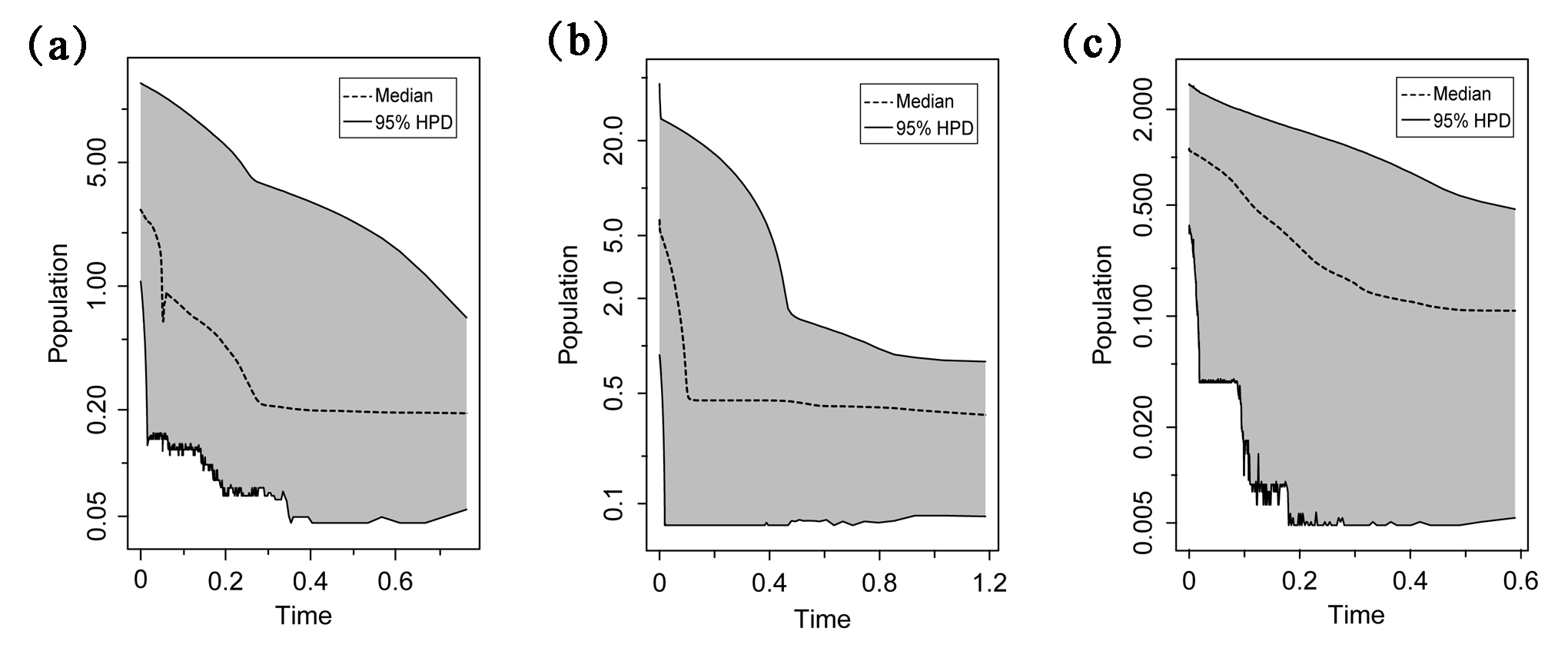


**Fig. S4** BEAST-derived extended Bayesian skyline plot for (a) *Machilus thunbergii* as a whole, (b) northern cluster, and (c) southern cluster. The median value and 95% high posterior density (HPD) intervals are shown.


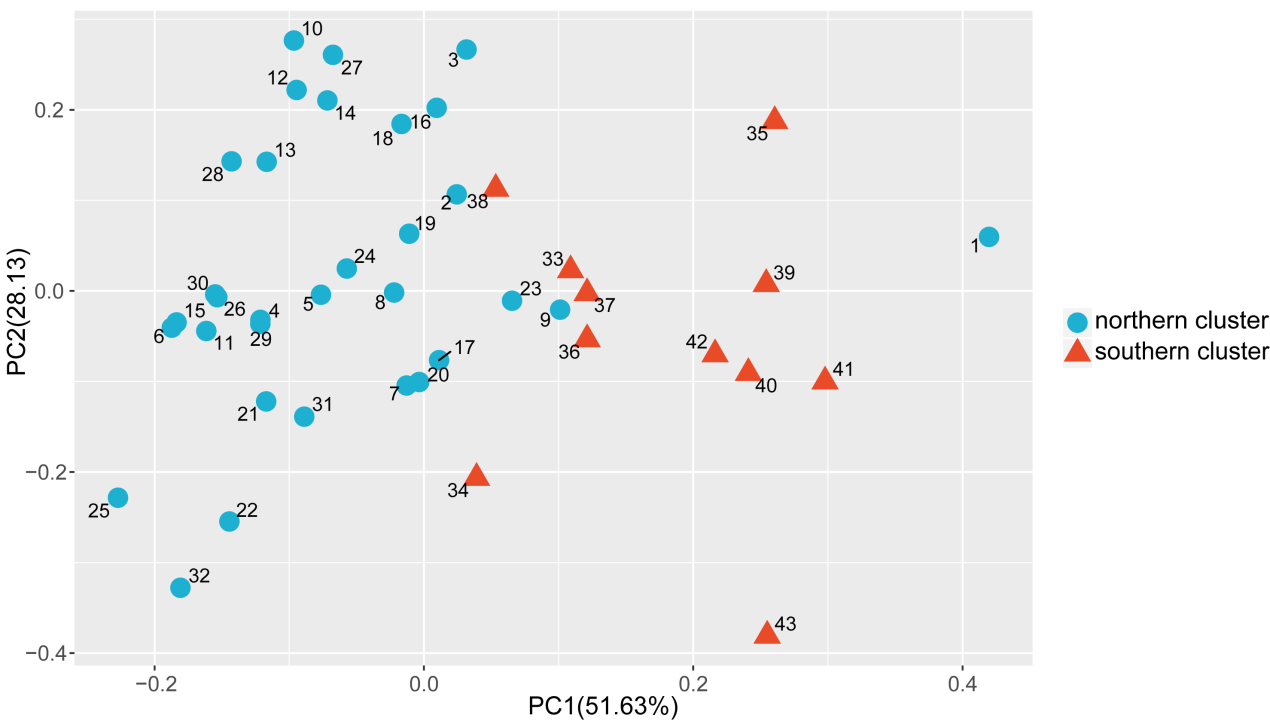


**Fig. S5** Principal component analysis (PCA) plots of the first two components of climate data from the sampling sites of the northern and southern clusters of *M. thunbergii.*

**
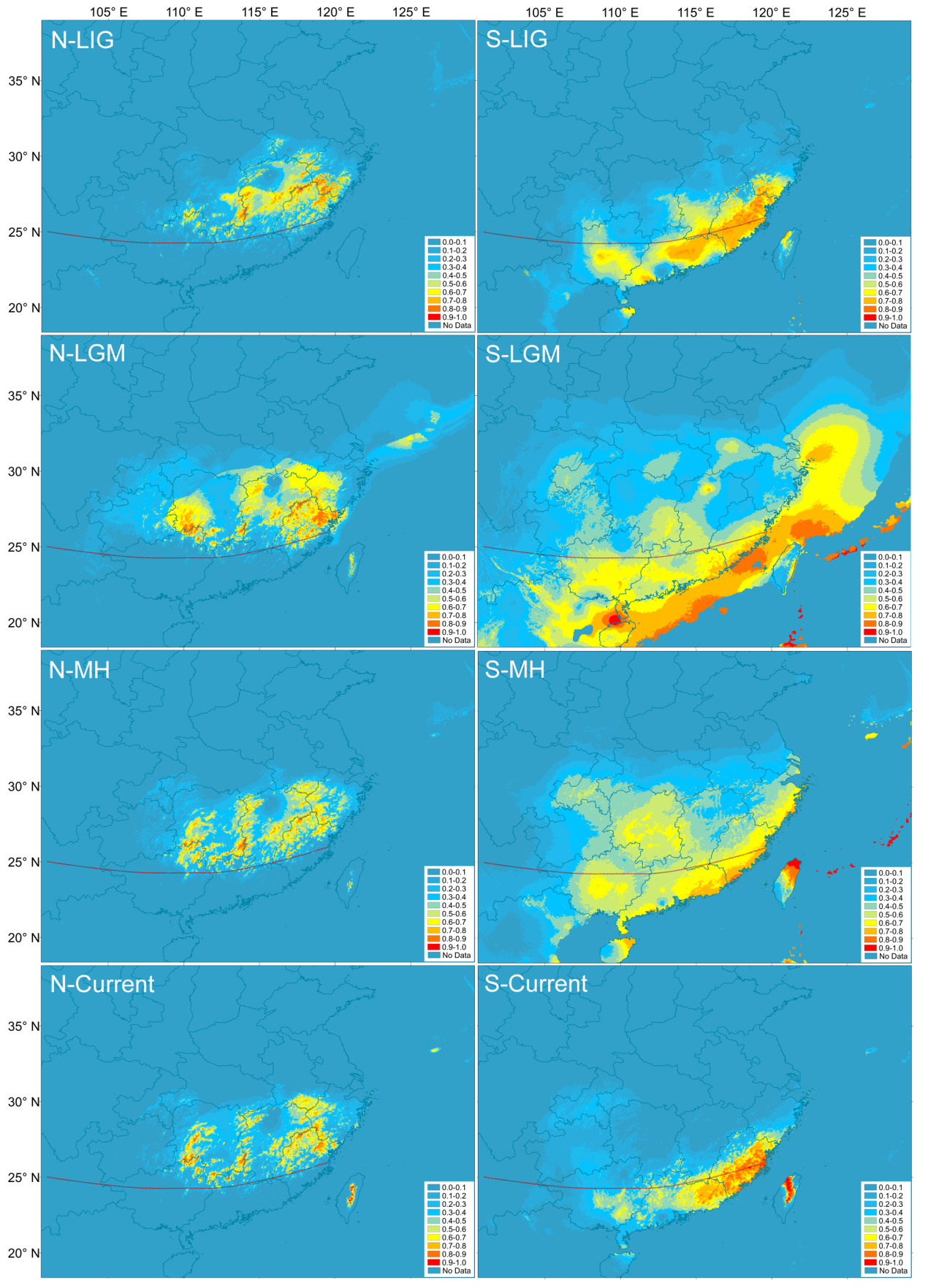
**

**Fig. S6** Potential distributions of *M. thunbergii* groups (N, S) for the last interglacial (LIG), the last glacial maximum (LGM), the middle Holocene (MH) and the present time (current).
